# Systems biology and microbiome innovations for personalized diabetic retinopathy management

**DOI:** 10.1038/s41540-025-00607-w

**Published:** 2025-11-21

**Authors:** Javad Aminian-Dehkordi, Fateme Montazeri, Ali Tamadon, Mohammad R. K. Mofrad

**Affiliations:** 1 Molecular Cell Biomechanics Laboratory, Departments of Bioengineering and Mechanical Engineering, University of California, Berkeley, CA USA; 2 Department of Ophthalmology & Vision Science, Tschannen Eye Institute, University of California, Davis, Sacramento, CA USA; 3 Molecular Biophysics and Integrative Bioimaging Division, Lawrence Berkeley National Lab, Berkeley, CA USA

**Keywords:** Metabolic engineering, Computational biology and bioinformatics, Microbiology, Systems biology, Molecular medicine, Pathogenesis

## Abstract

Diabetic retinopathy (DR), a complex condition driven by inflammation, oxidative stress, and metabolic imbalances, calls for innovative treatment strategies. Engineered probiotics delivering angiotensin-converting enzyme 2 (ACE2) offer a promising strategy by leveraging gut microbiome-retina association. Advances in synthetic biology and computational techniques enable personalized, data-driven therapies. This review discusses computational approaches at multiple scales and presents an integrated framework for promoting personalized, systems-level DR management.

## Introduction

Diabetic retinopathy (DR) is a major sight-threatening complication of diabetes and a leading cause of vision loss worldwide^1^. Conventional treatments, such as controlling blood sugar levels and ocular procedures, can slow its progression but do not target its systemic causes. Increasing evidence underscores the impact of gut microbiome imbalance on inflammation, oxidative stress, and metabolic regulation, which are crucial in DR^2–4^. This concept, known as the “gut-retina axis,” offers new possibilities for therapeutic intervention^5^. Table S1 lists important factors related to DR pathogenesis, as reported in the literature.

Among systemic modulators, angiotensin-converting enzyme 2 (ACE2) has become a molecule of interest due to its role in maintaining vascular health and regulating inflammation^6^. While ACE2 has been studied in conditions such as cardiovascular disorders^7^, pulmonary arterial hypertension^8^, and myocardial ischemia-reperfusion injury^9^, its specific involvement in DR presents a unique upstream approach to restore balance in retinal and systemic pathways.

Synthetic biology offers innovative strategies for gut microbiome modulation. Engineered probiotics can be designed to deliver therapeutic molecules, enhance gut functions, and modulate host-microbe interactions related to DR^10^. Parallel advances in gene circuits, CRISPR-based editing, and bioencapsulation further expand the possibilities for targeted microbiome engineering^11,12^. However, ensuring precision, stability, and safety remains a challenge for clinical translation^13^.

Computational tools can support the design and prediction of therapeutic efficacy. Multi-omics integration, machine learning (ML), and biomechanical simulations provide systems-level insights into host–microbe interactions and help optimize engineered probiotics for therapeutic use.

In this review, we synthesize current knowledge on the ACE2 modulation through the gut microbiome and highlight how synthetic biology, computational systems biology, and artificial intelligence (AI) can converge to inform next-generation DR therapies. By connecting mechanistic insights with data-driven modeling, we present tools for personalized, microbiome-based interventions that advance conventional treatment approaches.

## ACE2 and microbiome modulation in DR pathophysiology

*RAS in DR*. The renin-angiotensin system (RAS) is a complex hormonal cascade primarily involved in regulating blood pressure, electrolyte homeostasis, and fluid balance; however, its dysregulation contributes to retinal microvascular dysfunction^14^. Elevated levels of angiotensin II (Ang2), a key RAS effector, exert vasoconstrictive, pro-inflammatory, and pro-oxidative effects within the retina^15^. These actions promote endothelial dysfunction, increase vascular permeability, and induce cytokine and chemokine production, which activate leukocytes and amplify vascular endothelial growth factor (VEGF) signaling^16,17^. Together, these processes lead to ischemia, vascular leakage, and diabetic macular edema.

Pharmacological inhibition of the RAS through angiotensin-converting enzyme (ACE) inhibitors or Ang2 receptor blockers (ARBs) can decrease inflammation and oxidative stress, which results in improved clinical outcomes in patients with DR. Therefore, targeting the RAS is a promising approach for managing DR and its related complications^18^.

**Fig. 1:**
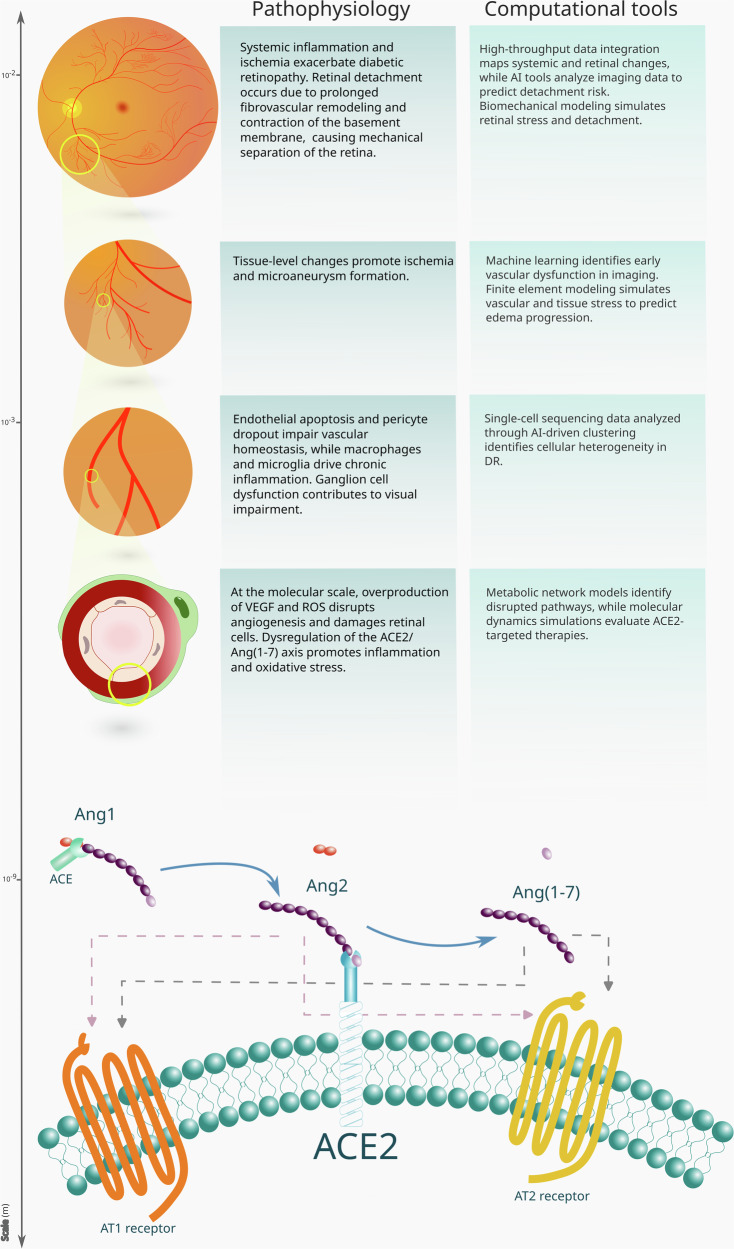
Computational and molecular perspectives on ACE2 in diabetic retinopathy. (top) Overview of the application of computational methods on diabetic retinopathy at different scales, with a focus on ACE2; (bottom) the renin-angiotensin system is upregulated during inflammatory states, resulting in increased synthesis of Ang2. This heightened production of Ang2 can happen both systemically and locally within organs, inducing vessel vasoconstriction. ACE2 can degrade Ang2 (ACE2, angiotensin-converting enzyme 2; Ang2, angiotensin II; RAS, renin–angiotensin system).

**Fig. 2:**
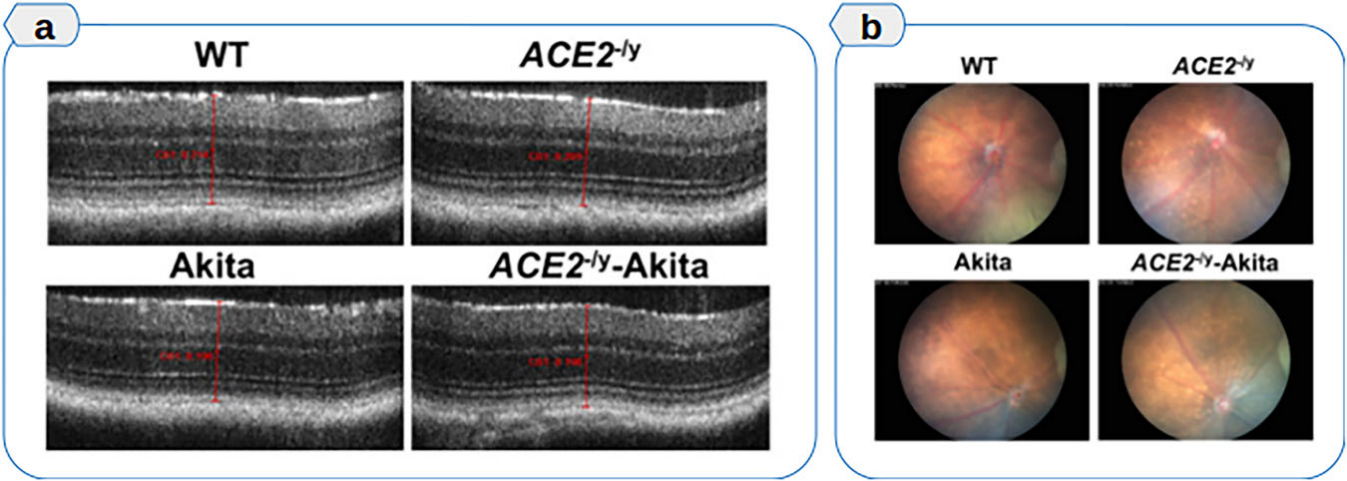
Impact of ACE2 deficiency on retinal structure and pathology in diabetic mice. **a** Representative OCT images showing reduced retinal thickness in diabetic cohorts (Akita and ACE2^–/y^-Akita groups) compared to wild-type and ACE2^–/y^ groups. Quantification revealed a reduction in retinal thickness associated with diabetes, independent of ACE2 loss. **b** Fundus images illustrating a marked increase in white retinal lesions in ACE2^–/y^ mice, indicative of retinal nerve fiber layer infarcts. These changes occurred regardless of diabetic status, implicating ACE2 deficiency as the primary driver (figure adapted with permission from^22^) (OCT, optical coherence tomography; ACE2–/y, ACE2 knockout mice).

*ACE2 in DR*. Beyond RAS blockade, attention has shifted toward ACE2^19^, a protective factor that degrades Ang2 to produce angiotensin-(1–7) (Fig. 1)^20^. Reduced ACE2 activity in diabetes can disrupt this balance, favoring Ang2-mediated damage. Unlike ACE inhibitors or ARBs, which suppress Ang2 activity, targeting ACE2 restores this equilibrium^21^. Studies indicate that ACE2 deficiency exacerbates diabetes-induced vascular dysfunction and worsens DR, while Ang(1–7) supplementation restores impaired functions of bone marrow–derived CD34+ (Fig. 2)^22^. These findings highlight the therapeutic potential of the ACE2/Ang(1–7) axis.

However, systemic modulation of ACE2 is complicated. Over- or under-activation of ACE2 can disrupt the Ang2/Ang(1–7) balance, with systemic effects on blood pressure and other organs. Moreover, systemic delivery may not provide enough retinal concentrations and could cause off-target effects. The blood-retina barrier limits the entry of ACE-modulating agents into the retinal tissue, making effective delivery more difficult^23,24^. Another challenge arises from the interconnected processes underlying DR, where multiple pathological mechanisms contribute to its progression. While ACE2-based interventions may address inflammation, they do not directly counteract oxidative damage or neural degeneration^25,26^. Also, interindividual variability in ACE2 expression, affected by genetic and environmental factors^27^, further complicates the development of a one-size-fits-all therapeutic strategy.

These limitations have shifted research interests toward the gut microbiome, which can modulate ACE2 expression and activity through the gut-retina axis. Microbiota modulation offers an indirect yet systemic route to improve ACE2 regulation and provide complementary therapeutic benefits.

## Regulation of ACE2

The gut microbiome and RAS engage in a dynamic, bidirectional interplay essential for homeostasis^28^. Microbial metabolites, such as short-chain fatty acids (SCFAs) and other bioactive compounds, modulate systemic RAS activity, influencing inflammation, vascular function, and immune regulation. Conversely, RAS dysregulation can alter the gut environment, impacting microbiota composition and metabolic activity ^29^. This connection has prompted the exploration of microbiome-based interventions, such as probiotics and dietary supplements, which target upstream mechanisms of DR. These non-invasive solutions offer systemic benefits by modulating enzyme activity, including ACE2, across multiple tissues.

ACE2 serves as a critical link in this interaction. Predominantly expressed in gut epithelial cells, ACE2 regulates RAS by converting Ang2 into Ang(1–7), while also maintaining dietary amino acid transport, particularly tryptophan, and preserving gut barrier integrity ^30–32^. Dysregulation of ACE2 in the gut can cause systemic inflammation, increased intestinal permeability, and metabolic disturbances, all of which indirectly contribute to DR^33,34^. By modulating ACE2 expression, the gut microbiome emerges as a potential upstream regulator of both gut and systemic health (Box 1).

Box 1 From concern to opportunity The SARS-CoV-2 pandemic drew significant attention to ACE2, the viral entry receptor, and raised initial concerns about the safety of ACE2 therapies. However, this global focus also accelerated research into its physiological functions, revealing more about its roles in vascular and inflammatory diseases, including DR. Therapeutic strategies targeting ACE2 offer local benefits in the retina and systemic protection against diabetes-associated vascular injury in other organs. This dual action has made ACE2 an attractive target for integrated management of diabetic complications^22,128,129^.

## Engineering probiotics for human ACE2 delivery

**Fig. 3:**
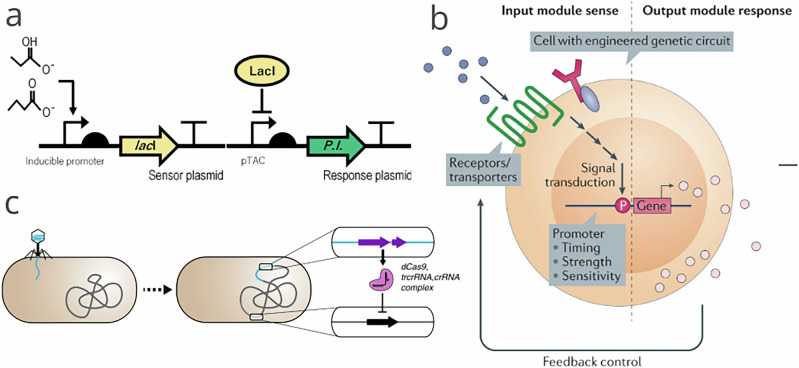
Synthetic biology strategies for programmable microbiome therapeutics. This figure highlights examples of engineered approaches for microbiome-based therapy. **a** Metabolite-responsive genetic circuits. SCFA-sensing NOT-gate circuit in *E. coli* responds to propionate or butyrate and dials down a transgene, e.g., sfGFP or mGM-CSF. This leads to an inverse output at high SCFA levels (figure adapted from^124^, licensed under CC BY 4.0). **b** Design logic for living therapeutics. Disease-associated biomarkers are detected and processed by genetic circuits, and signals are then converted into programmed responses, such as therapeutic production, secretion, degradation, or surface display, with tunable feedback control (figure adapted with permission from^125^). **c** In-gut CRISPR-based modulation. Modulation of bacterial genes in the mammalian gut using encapsulated bacteriophages carrying CRISPR/dCas9 machinery. This enables targeted control of colonizing strains (figure adapted from^126^, licensed under CC BY 4.0) (SCFA, short-chain fatty acid; GFP, green fluorescent protein; mGM-CSF, murine granulocyte–macrophage colony-stimulating factor; CRISPR, clustered regularly interspaced short palindromic repeats).

To address the challenges associated with ACE2 regulation, synthetic biology offers strategies (see Box 2), including the design and development of engineered probiotics for targeted ACE2 delivery (Fig. 3). Safe probiotic strains can serve as live vectors for the oral administration of ACE2, engineered to enhance tissue bioavailability. This approach harnesses the interplay between the systemic and the tissue-specific RAS networks that collectively maintain physiological homeostasis.

**Fig. 4:**
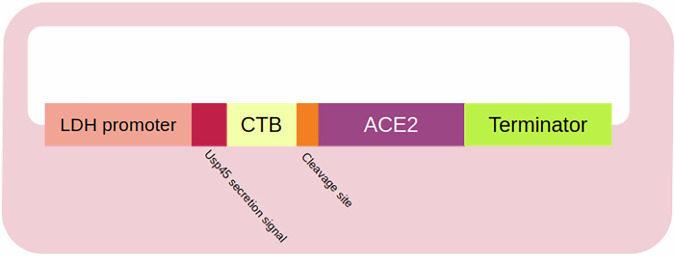
Engineered *Lactobacillus* vector for ACE2 expression. This figure illustrates a probiotic construct designed to express codon-optimized human ACE2^127^. The construct consists of the LDH promoter, a strong constitutive promoter derived from the lactate dehydrogenase gene of *Lactobacillus acidophilus*, which drives the expression of the gene cassette, starting with a signal peptide sequence from the Usp45 protein and CTB for enhanced stability. ACE2 is expressed as a secreted fusion protein alongside CTB. A furin cleavage site is positioned between ACE2 and CTB, which facilitates the release of ACE2 upon expression. The transcriptional terminator sequence ensures proper termination of mRNA synthesis (ACE2 angiotensin-converting enzyme 2, LDH lactate dehydrogenase, CTB cholera toxin subunit B).

*Engineered probiotic platforms*. *Lactobacillus paracasei* has emerged as a leading candidate for recombinant probiotic delivery of human ACE2^35^. A ubiquitous member of the gut microbiome, *L. paracasei* has been shown to strengthen barrier integrity by upregulating tight junction proteins, and has demonstrated clinical benefit in conditions such as diarrhea and irritable bowel syndrome^36^. These properties make it a versatile platform for oral delivery, offering both localized gastrointestinal effects and systemic therapeutic potential. For detailed examples of engineered *L. paracasei* constructs designed for ACE2 delivery, see Box 3.

Box 2 Programmable microbial therapies for DR Synthetic biology enables programming of biological entities to produce therapeutic molecules, repair damaged tissues, or modulate pathways for medical applications^130^. Unlike conventional DR treatments that mainly manage symptoms, programmable microbial therapies target metabolic and inflammatory mechanisms underlying disease (Fig. 3).

*Synthetic gene circuits*. These circuits can be integrated with cells and regulate therapeutic gene expression in response to disease-relevant cues^131^. In the gut, they sense inflammatory signals and trigger counteractive molecule production^12,132^. Designer mammalian cells with glucose-responsive circuits exemplify precision control^133–135^, though challenges include specificity and sensitivity, off-target effects, and preventing horizontal gene transfer^136,137^.

*Microbiome editing*. CRISPR-based tools can reprogram gut microbes by silencing pathogenic genes (CRISPRi) or enhancing beneficial traits (CRISPRa). Proof-of-concept studies have selectively eliminated antibiotic-resistant bacteria, showing potential for suppressing pro-inflammatory microbes in DR^138^. However, safety, immune responses to CRISPR components, and ethical concerns remain^139^.

*Therapeutic molecule production*. Gut microbes can be engineered as in situ fermenters to produce anti-inflammatory metabolites. Examples include SCFAs, *e.g*., butyrate^124,140^, and tryptophan-derived metabolites, such as indole-3-aldehyde, which modulate the aryl hydrocarbon receptor pathway and angiogenesis^141^. Individual variability in microbiota and achieving therapeutic concentrations are key limitations.

*Bioencapsulation and targeted delivery*. These methods protect probiotics during gastrointestinal transit and release them at target sites in response to pH or inflammatory markers^142^. Recent innovations in polymer- and hydrogel-based microcapsules provide precise spatiotemporal control over their release^143^.

*Engineered probiotics*. Probiotics can be engineered to secrete anti-inflammatory molecules or antioxidants that mitigate oxidative stress and inflammation^144–146^. These versatile platforms could be tailored to retinal disease pathways in DR^142,147^.

Box 3 *Lactobacillus paracasei* as a vector for ACE2 delivery Figure 4 shows the engineering of a *Lactobacillus* vector designed for ACE2 expression, starting with a shuttle plasmid containing a green fluorescent protein (GFP) reporter gene. To address limited ACE2 expression in *L. paracasei*, three codon-optimized variants of the ACE2 gene were developed, replacing the GFP gene in the plasmid. Among these, one construct exhibited significantly elevated enzymatic activity and was selected for further development. The optimized ACE2 was engineered for secretion and fused with cholera toxin B to enhance transmucosal transport into the bloodstream. When tested in mouse models, oral administration of *L. paracasei* carrying this construct increased ACE2 levels in serum and tissues, effectively mitigating diabetes-induced retinal damage, highlighting its therapeutic potential^127^.

A complementary study investigating *L. paracasei* examined whether mitigating imbalances in intestinal RAS could improve gut barrier dysfunction and prevent DR progression^148^. The study demonstrated that *L. paracasei*-ACE2 administration over three months enhanced retinal function and visual acuity in Akita-ACE2 mice relative to Akita mice with a six-month history of type 1 diabetes. This investigation was shown to upregulate intestinal ACE2 expression, diminish Ang2 and ACE levels, and increase gut barrier integrity in Akita-ACE2 mice. These outcomes were attributed to enhanced ACE2 bioavailability. Subsequent research revealed that *L. paracasei*-ACE2 administration restored intestinal lacteal function, reinforced both gut and retinal barrier integrity, and reduced DR severity in Akita mice, further supporting the potential of probiotics targeting the gut-retinal axis as a therapeutic modality for the management of DR^149^. It is important to note that this evidence is derived from preclinical mouse models; the transition to human clinical trials will be essential to validate both the safety and efficacy of this engineered probiotic approach in patients.

Box 4 Other regulators of the gut-retina axis Beyond omics data, non-coding RNAs, such as micro RNAs (miRNAs) and small RNAs (sRNAs), play roles in mediating host-microbe interactions. These molecular entities have a dual impact on gut microbial homeostasis and retinal inflammation. miRNAs, for instance, are increasingly recognized as modulators of immune signaling and inflammatory cascades, both of which are fundamental to DR pathogenesis^150^, and their expression is in turn modulated by gut microbes, with downstream effects on systemic inflammation^151^. Similarly, microbial sRNAs are emerging as potent signaling molecules that may exert systemic effects either directly through systemic circulation or indirectly by modulating host immune and metabolic networks^152^. These findings highlight the potential of RNA-centric regulatory mechanisms as therapeutic targets in the gut-retina axis.

Box 5 GEMs in synthetic biology GEMs play a complementary role in the design and simulation of synthetic circuits for engineering probiotics. They help predict the metabolic impact of introducing synthetic circuits into a host organism^153^. This ensures that synthetic gene circuits are designed with minimal disruption to the native metabolism while achieving the desired functionality. GEMs have been reported to be useful in developing microbial strains capable of producing pharmaceuticals, biofuels, and other valuable chemicals by identifying optimal pathways and resolving metabolic bottlenecks^154,155^.

One key application is optimizing resource allocation between the native metabolism and synthetic circuit operation^156,157^. GEMs can simulate how introducing a circuit for therapeutic molecule production affects the cellular energy budget and precursor metabolite availability. Studies have demonstrated that coupling GEMs with synthetic circuit design tools enables the identification of metabolic trade-offs and potential bottlenecks, highlighting how the metabolic steady state and transient response depend on regulatory topology and design parameters^158^.

GEMs can be integrated with dynamic modeling tools such as iBioSim to predict the interaction between a synthetic circuit and the host’s metabolic network under specific environmental conditions, such as pH, nutrient availability, or the presence of gut-specific substrates^159^. For instance, in designing a probiotic strain to release bioactive peptides in the gut, GEMs help identify the metabolic costs of peptide production and guide the placement of regulatory elements in the circuit for condition-specific expression.

Box 6 Integration of metabolic networks with multi-omics data (i) *Enhancing predictive power with host multi-omics data*: The integration of GEMs with host multi-omics data facilitates the identification of biomarkers and therapeutic targets related to ACE2 regulation. For example, combining GEMs with gene expression data^160,161^ can clarify how genetic variations influence the metabolic pathways governing ACE2. In this regard, Table S2 represents some tools used to integrate gene expression data into GEMs.

(ii) *Addressing dysregulation of microbial metabolites*: While meta-omics analyses can establish correlations between microbial abundances and metabolite profiles, the underlying causal pathways often remain unclear^162^. Omics data alone cannot resolve which microbial or host metabolic processes are driving these changes. Integration with GEMs addresses this gap by providing a network-based, mechanistic framework^163^. For example, GEMs can model the impact of dietary inputs on microbial metabolic pathways and predict downstream effects on metabolite levels and their potential implications for host health. This capability is particularly valuable in diseases where both microbial and host factors shape the metabolome. By simulating these interactions, GEMs move beyond static correlation to provide mechanistic explorations for observed changes.

(iii) *Metagenome-scale modeling (MSM)*: MSM extends GEMs by integrating multi-omics data to capture the intricate metabolic interactions within microbial communities, such as those in the gut microbiome^164^. MSM supports reactobiome analysis, which elucidates metabolic capabilities, cross-species interactions, and links community-level processes to host health^165^. It can also simulate metabolite exchange dynamics and predict the effect of interventions, such as probiotic supplementation or dietary changes, on community structure and metabolic outputs^80^. Despite its transformative potential, MSM faces challenges, including incomplete metagenomic data, computational complexity, and the need to incorporate dynamic and spatial features of communities. Advances such as single-cell metagenomics and AI-driven model optimization are expected to refine MSM to boost its translational potential.

Box 7 Risks of ACE2 overexpression *Viral infection risk*. ACE2 is the primary entry receptor for SARS-CoV-2, and its expression influences infection risk^166,167^. High ACE2 levels in nasal and alveolar epithelial cells facilitate viral entry. Intestinal enterocytes also serve as susceptible targets, consistent with gastrointestinal symptoms of COVID-19^168^. While the gut is not considered the major portal of entry compared to the respiratory tract, elevated intestinal ACE2 could expand the pool of target cells. Supporting this, cells with little endogenous ACE2, such as vascular endothelial cells, become infectable and mount strong inflammatory responses once ACE2 is overexpressed. Thus, although restoring ACE2 activity may protect against diabetes and DR, unchecked overexpression could increase vulnerability to viral infection.

*Gastrointestinal risk*. While deficiency is often implicated in DR, overexpression in the gut may also be harmful. In Crohn’s disease, markedly elevated colonic ACE2 expression was associated with a more than two-fold higher risk of surgery within five years^169^. Excessive ACE2 expression is not uniformly protective and may reflect or promote a pathological state. Any therapeutic strategy to enhance ACE2 must therefore balance systemic vascular benefits against potential risks of aggravating intestinal disease in susceptible individuals.

Box 8 Translational pathway of engineered probiotics While engineered probiotics offer promising preclinical evidence in DR, translation to clinical practice remains an emerging area: current data are encouraging but preclinical, and to date, no interventional DR trials of engineered probiotics have been registered. Nevertheless, human experience with engineered live biotherapeutic products (LBPs) in other diseases and recent approvals of microbiota-based (non-engineered) therapies suggest growing regulatory familiarity with LBPs. For instance, *E. coli* Nissle engineered for phenylketonuria has shown safety and pharmacodynamic activity in Phase ½ studies^170,171^.

Regulatory and safety considerations are the primary hurdles for translation. In the United States, LBPs are developed under Investigational New Drug (IND) applications, which require detailed Chemistry, Manufacturing, and Control (CMC) documentation, including strain identity, potency, genetic stability, and absence of contaminants^172,173^.

For DR, first-in-human studies will likely enroll patients with non-proliferative DR, where efficacy can be tested with sensitive imaging endpoints, such as OCT-angiography and paired systemic biomarkers. Computational tools can help optimize strain design, anticipate metabolic burden, and identify biomarkers linking gut activity to retinal outcomes. Addressing regulatory requirements and biocontainment safeguards could provide a path for translating engineered probiotics from preclinical promise to clinical evaluation in DR.

### Barriers in probiotic therapeutics

Despite the promise, several challenges limit the translation of engineered probiotics into clinical therapies. One of the primary challenges is therapeutic dosage control: unlike conventional protein drug delivery systems, which can be timed to reach peak concentrations in the body, probiotics continuously secrete therapeutic proteins. As a result, the effective dosage depends on several factors beyond just protein expression levels and the initial bacteria dose^37^. Key among these variables is the survival and persistence of orally administered probiotics within the gastrointestinal tract. This variability complicates optimization of therapeutic outcomes.

Another unresolved challenge is understanding the broader biological impact of introducing recombinant strains into the human microbiome. Engineered bacteria may alter gut community composition or immune responses in unpredictable ways, particularly in patients with underlying health conditions^38^. Careful investigation into host–microbe interactions, stability of engineered strains, and potential off-target effects will be critical to ensuring safety. Targeted editing of gut microbes perturbs specific functions while largely preserving community composition, aiding safety evaluation.

Despite these challenges, engineered probiotics represent a promising delivery system for ACE2 and other therapeutic proteins. The success of such therapies hinges on overcoming these limitations through targeted research and development in synthetic biology, with the goal of optimizing the stability, safety, and efficacy of probiotic-based treatments.

## Computational modeling in DR and microbiome research

**Fig. 5:**
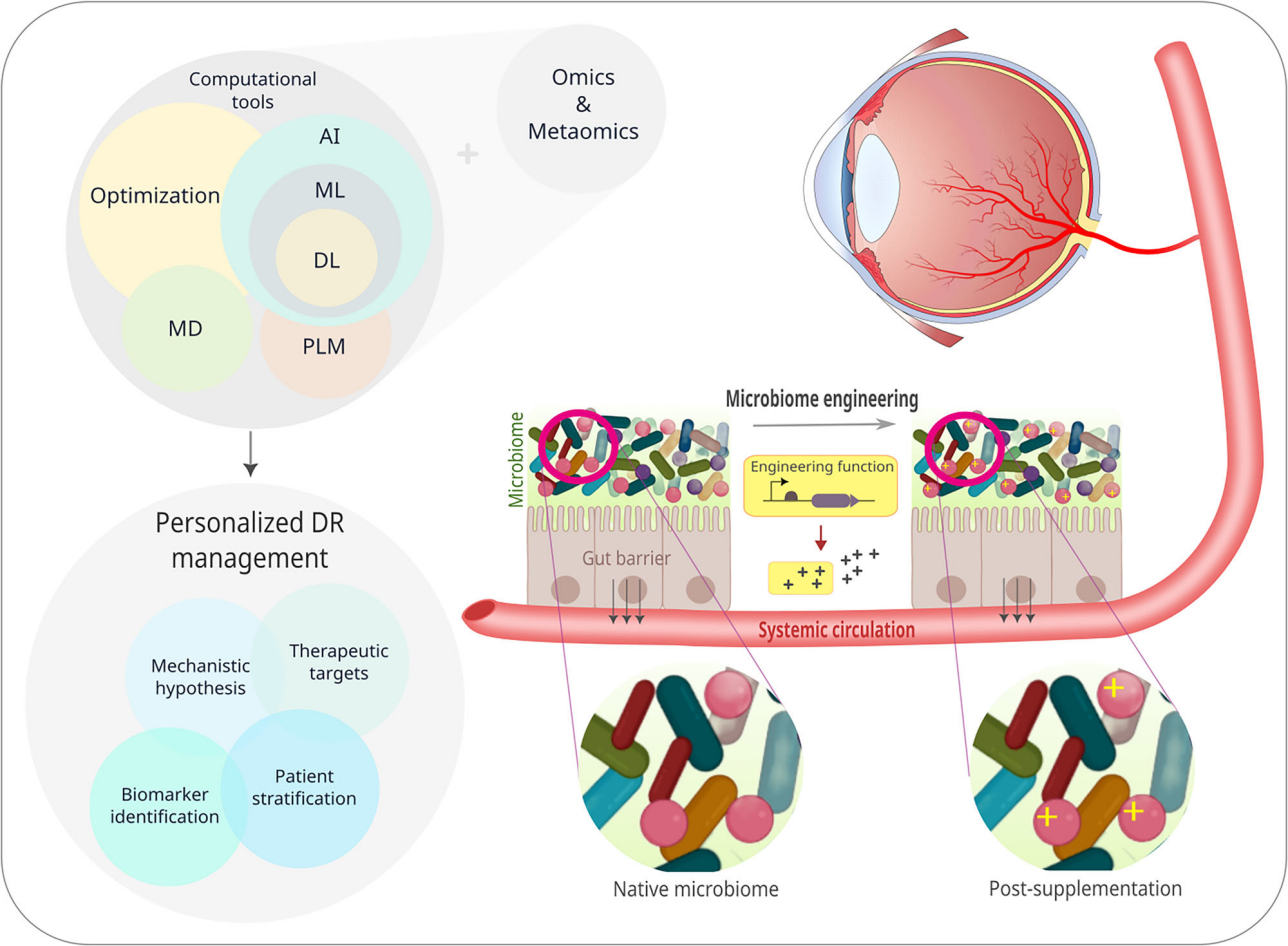
Computational approaches for advancing microbiome- and ACE2-targeted therapies in diabetic retinopathy. Synthetic biology approaches, such as engineered probiotics, together with computational frameworks, enable the design and validation of next-generation therapeutic interventions (DR diabetic retinopathy, AI artificial intelligence, ML machine learning, DL deep learning, MD molecular dynamics, PLM protein language model).

To address existing challenges, computational methods have become powerful tools. These approaches streamline the design and evaluation of engineered probiotics, predict survival and colonization dynamics, and simulate host-microbe interactions and metabolite production. More broadly, computational models integrate multi-omics datasets, identify microbial genes and pathways influencing key regulators such as ACE2, and optimize synthetic constructs for targeted interventions. In this way, systems-level computational tools provide a foundation for rational probiotic engineering, predictive biomarker discovery, and mechanistic insight into how gut microbiota influence diabetic retinopathy (Fig. 5).

## High-throughput omics technologies

Advances in genomics, transcriptomics, proteomics, and metabolomics have significantly deepened our understanding of the gut microbiome and its systemic impacts, including retinal health. By linking gut microbial diversity to host immune responses and metabolic pathways, these technologies can provide mechanistic insights into the microbiome’s role in disease pathogenesis (see Box 4).

### Decoding microbial composition and function

Metagenomic sequencing enables the identification of microbial taxa and gene repertoires associated with key metabolic and inflammatory pathways relevant to DR. Genes encoding enzymes for pro-inflammatory mediators or anti-inflammatory metabolites, such as SCFAs, provide mechanistic links between gut dysbiosis and retinal injury^39^. Shotgun sequencing offers a broader view of microbial functional capacity, but interpretation is limited by its static nature^40^. Complementary metatranscriptomics adds a dynamic perspective and provide real-time insights into active microbial gene expression^41^. Such analyses reveal the temporal and contextual activity of microbial genes and further our understanding of the gut microbiome’s role in systemic inflammation and retinal disease progression^42^.

### Bridging metabolism to retinal pathophysiology

Metabolomics, in particular, provides a tool for profiling gut microbiota-derived metabolites and determining their impact on host metabolic states *via* mass spectrometry and nuclear magnetic resonance (NMR) spectroscopy. Changes in key microbial metabolites, including SCFAs, bile acids, and tryptophan derivatives, have been documented in DR patients, with significant correlations to disease severity and progression^43,44^. In efforts to explore the effect of dysbiosis in disease exacerbation, metabolomic analyses in proliferative DR revealed reduced microbial diversity and disrupted metabolite-host interactions^39,45^. These insights can hint at biomarkers but require validation across cohorts due to high interindividual variability. Proteomics extends this view by mapping host pathways impacted by microbial products, including those linked to inflammation and other DR-related processes. Dysregulation of proteins involved in immune signaling and angiogenesis has been reported in DR, with evidence suggesting modulation by the gut microbiota^46,47^. In parallel, transcriptomic analyses of retinal tissues reveal differential gene expression patterns that link metabolic disturbances to gut microbial dysbiosis^48,49^. Such integrative analyses highlight the complex molecular dialog between gut microbes and the retina.

## Multi-omics data and AI

Integrating multi-omics data provides an understanding of how microbial diversity, metabolite production, and host inflammatory pathways interact in DR. Advanced bioinformatics and AI approaches provide the computational power to synthesize and analyze these large datasets, uncover novel biomarkers, and identify therapeutic targets. Such integrative approaches, discussed in the following, are pivotal for advancing precision medicine in DR or other systemic diseases.

### AI-enabled microbiota analysis

Machine learning (ML) and deep learning (DL) are transforming our ability to decode complex host–microbe relationships and profile the gut microbiome and its metabolites. Such approaches are particularly relevant in managing conditions like DR, where gut microbiota dysbiosis is increasingly recognized as a contributing factor^50,51^.

ML has proven instrumental in integrating diverse data types to identify molecular signatures associated with disease states. Supervised techniques like random forests and support vector machines excel in predictive modeling^52–54^, while unsupervised clustering methods reveal previously unrecognized microbial patterns^55^. For instance, studies integrating multi-omics data, including metagenomic and metabolomic profiles, have successfully stratified patients by microbial species and metabolic pathways linked to inflammatory and metabolic disorders^56^. More advanced methods like partial least squares-discriminant analysis (PLS-DA) further enhance biomarker discovery, with applications in identifying metabolic signatures relevant to DR^57^.

DL extends this capability by capturing highly non-linear relationships in microbiome data. Convolutional neural networks have been applied to phenotype prediction^58^, while graph neural networks (GNNs) model hierarchical and functional interactions within microbial communities^59,60^. Recent work shows the ability of GNNs to integrate multi-omics microbiome datasets and predict host phenotypes associated with inflammatory conditions^61^. This graph-based perspective is relevant to the gut-retina axis, where interactions among microbial species, metabolites, and host pathways may drive DR progression.

The utility of a GNN is fundamentally dependent on the underlying graph structure. Many early applications relied on simple correlation-based graphs, which may not reflect actual biological mechanisms. New computational frameworks use graph-based approaches to bridge mechanistic predictions with deep learning. For instance, SIMBA provides a scalable framework for integrating metabolic-based interaction graphs with a custom graph transformer^62^. Such approaches show how AI can be trained on biological first principles rather than just statistical association.

### AI in probiotic research

Probiotic research has greatly benefited from ML and DL advancements, particularly in strain identification, functional prediction, and efficacy optimization. Platforms like iProbiotics employ ML algorithms to rapidly identify probiotic strains based on genomic and phenotypic data, streamlining the discovery and development process^63^. DL models have been influential in predicting functional traits of gut microbiota, such as the production of reactive oxygen species (ROS) scavenging enzymes, which are crucial for mitigating oxidative stress^64^. Time-series models, including recurrent neural networks (RNNs), can be used to evaluate the longitudinal impact of probiotics on gut microbial composition and systemic health^65^. This kind of research enables the optimization of probiotic formulations for specific therapeutic outcomes.

### Limitations of AI/ML models

Microbiome datasets are relatively small in size and heterogeneous in quality, and this makes them prone to overfitting with less generalizability across populations. Many algorithms lack interpretability, which constrains clinical trust and adoption, particularly when it comes to therapeutic design. Most models rely on cross-sectional data, whereas longitudinal datasets are essential for capturing dynamic host–microbe interactions relevant to DR. Validation of AI-informed predictions in clinical studies is still limited; this highlights the importance of translational pipelines before clinical applications. Emerging computational strategies are beginning to address these limitations.

With the rise of large language models (LLMs), LLM–powered agents are being developed to integrate heterogeneous microbiome datasets, guide hypothesis generation, and provide context-aware explanations for model predictions^66^. These advances hold promise for making AI-driven microbiome research both more reliable and clinically actionable.

## Metabolic networks and constraint-based tools

Microbial ecosystems are highly dynamic, with species engaging in complex cooperative and competitive metabolic interactions. Genome-scale metabolic models (GEMs) provide a systems framework to map metabolic capabilities from genomic sequences and predict phenotypic responses to perturbations. By linking genotypes to phenotypes, GEMs enable the design of microbial communities with tailored traits, guide therapeutic strategies, and support applications in metabolic engineering, systems biology, and precision medicine^67^ (see Box 5).

### GEM reconstruction and challenges

GEMs are reconstructed from genomic sequences using annotation-based methods to define metabolic networks. Automated pipelines expedite this process^68,69^; however, incomplete or fragmented genomes, common in metagenome-assembled data, can induce gaps and inaccuracies^70,71^. To address this, recent methods like the *pan-Draft* approach^72^, GECKO toolbox^73^, CLOSEgaps^74^, and ModelSEED *v*2^75^ incorporate evidence from multiple genomes to improve accuracy. Still, manual curation along with detailed experimental data are essential to ensure reliable reconstructions^76,77^.

### Constraint-based models

A core method within GEMs is flux balance analysis (FBA), which applies linear programming to predict steady-state metabolic fluxes, often optimizing for an objective function, such as biomass production^78^. In microbial communities, this technique oversimplifies the complexities of interspecies interactions and must be adapted to account for the metabolic crosstalk among community members. Extensions like OptCom^79^ and MICOM^80^ address this by incorporating multi-level optimization strategies to balance individual- and community-level objectives. More recent adaptations, including community FBA (cFBA)^81^ and SteadyCom^82^, enforce synchronized growth rates across community members and improve predictions of substrate utilization and metabolite exchange dynamics.

### Insights into ACE2 regulation through GEMs

Direct applications of GEMs to ACE2 regulation remain limited; however, these models can illuminate upstream metabolic contexts that shape enzyme expression and activity. By simulating fluxes through pathways involved in angiotensin peptide synthesis and related networks, GEMs can help predict how metabolic shifts might alter ACE2-relevant processes. While GEMs do not directly predict protein abundance, methods such as *Δ*FBA^83^, which integrate differential gene expression data with GEM constraints, can refine flux predictions and highlight dysregulated pathways in diseases. Integrating transcriptomic and proteomic data further improves model contextualization and can help nominate candidate targets for ACE2 modulation^84^ (see Box 6). GEMs can also explore drug interactions: for instance, simulating how ACE inhibitors or angiotensin receptor blockers influence RAS components and mapping these effects onto broader metabolic networks^85,86^.Beyond characterization, metabolic networks have been used to identify candidate drug targets by comparing structural similarities between human metabolites and chemical libraries^87^.

### Limitations of constraint-based models

Metagenome-assembled genomes are often incomplete, and gaps in annotation can propagate errors into metabolic reconstructions. Model assumptions can oversimplify the non-equilibrium nature of microbial communities. Microbe-host interactions add further complexity, as GEMs rarely capture immune responses, spatial heterogeneity, or signaling cascades. Importantly, predictions from GEMs must be experimentally validated, and this becomes increasingly demanding at a community scale. These challenges underscore the importance of integrating metabolic networks with complementary approaches and validating them in physiology-relevant systems.

## Molecular dynamics simulations

Molecular docking and molecular dynamics (MD) simulations are common biophysical computational tools that provide detailed insights into the structural dynamics and interactions within biomolecular systems. Docking, commonly used in virtual screening, provides a prediction of how a ligand binds to a protein’s binding site, resulting in static binding poses and associated binding affinities. MD simulations additionally model the physical movements of ions and molecules over time, generating dynamic trajectories and evaluating stability of protein-ligand complexes that capture quantitative information, such as Root-Mean-Square Deviation (RMSD), Root-Mean-Square Fluctuation (RMSF), material coefficients, thermodynamic and kinetic information of events, and specific intermolecular force contributions^88^. Together, these simulations can show how molecules, produced by engineered probiotics, interact with host proteins and receptors, such as ACE2 and G-protein-coupled receptors (GPCRs)^89^. By modeling these interactions, researchers can predict binding affinities, identify potential binding sites, and analyze the conformational changes that occur upon binding. This level of detail is important for designing probiotics that effectively modulate ACE2 activity. Crucially, these computational simulations not only generate powerful mechanistic hypotheses but are inherently predictive; their findings require following experimental validation through in vitro binding assays and testing in animal models to confirm biological relevance.

Docking has been extensively used to virtually screen large libraries of small molecule binding inhibitors between ACE2 and other biologically relevant receptor binding domains^88,90,91^. Predictions such as binding affinity, ligand poses, and protein-protein interactions can be utilized to identify microbial compounds and derived metabolites with potential to modulate pathways and mechanisms of interest to DR. To further understand such mechanisms, MD simulations have been used to investigate the binding and activation mechanisms of SCFAs on free fatty acid receptors (FFARs), specifically FFAR2 and FFAR3^92^. MD simulations can also be used to explore lipid-protein interactions, focusing on how membrane lipids influence the function of biological molecules^93^.

### Gut barrier and tight junctions

Maintaining gut barrier integrity is important for reducing systemic inflammation and preventing the translocation of harmful molecules into the host cardiovascular system^94^. MD simulations have been used to study conformational dynamics of tight junction proteins, such as claudins, and their role in regulating paracellular permeability^95,96^. These models highlight how microbial metabolites and signaling molecules affect pore size and stability and offer mechanistic explanations for barrier disruption or reinforcement. Such insights are directly relevant to probiotic-based strategies aimed at strengthening gut and retinal vascular barriers.

### Microbe-derived signaling molecules

MD approaches have also elucidated how microbial components, such as lipopolysaccharides (LPS), interact with immune receptors like Toll-like receptors (TLRs) and their ligands. Detailed analyses have shown the structural and molecular mechanisms underlying TLR activation and selectivity. Simulations of TLR4-MD2-(myeloid differentiation factor 2)-LPS complexes, for example, revealed important hydrophobic clusters within MD2 mediating receptor dimerization and activation^97^. Similar studies on TLR8 show how ligand selectivity fine-tunes immune responses^98^. These findings deepen understanding of how microbial molecules drive inflammatory cascades in DR and may inform rational design of probiotic metabolites or small molecules to modulate TLR signaling^99^.

### Quorum sensing and microbial balance

Quorum sensing (QS) is a bacterial communication mechanism through which bacteria coordinate gene expression based on their population density by producing, releasing, and detecting signaling molecules^100^. QS regulates various microbial functions, including biofilm formation, virulence, and competition between bacterial species^101,102^.

Probiotics can influence QS systems by disrupting pathogenic signaling or enhancing beneficial pathways^103^. Pathogenic bacteria often rely on QS to establish dominance within the gut, which results in dysbiosis^104,105^. On the other hand, probiotics and their metabolites can act as QS inhibitors; they interfere with pathogenic communication systems to restore microbial balance^106^. QS in commensal or probiotic bacteria also facilitates cooperative behaviors, such as nutrient sharing and synchronized biofilm formation, which collectively support gut integrity and function^107^.

Docking and MD simulations can characterize interactions between QS signals and regulatory proteins^108^. Extending these techniques to ACE2-related processes may aid in exploring ligand-protein complexes over time and also predicting drug-likeliness and ADMET profiling of candidate microbial compounds^109–111^.

### Limitations of molecular dynamics

Molecular docking’s primary limitation is that it simply scores the binding of rigid molecules using simplified criteria functions. This oversimplification can lead to inaccuracies in predicting the binding affinity between proteins and ligands from their true in vivo interactions. MD simulations are computationally expensive, typically limited to capturing nanosecond- to microsecond-scale dynamics on supercomputing clusters, whereas biological processes such as receptor activation and chronic inflammation occur over much longer timescales. Both methods are limited by the accuracy of approximate force fields, and solvent models may not fully replicate physiological environments.

Despite these limitations, proper use and integration of docking and MD can be powerful. Docking’s simplicity makes it fast, ideal for narrowing down large libraries of small molecules, while the more computationally intensive MD can be run on fewer systems. MD should be viewed as a powerful hypothesis-generating tool, best used in combination with experimental validation and systems-level modeling to extend findings to tissue-level outcomes.

## Protein structure prediction toolchains

Computational protein structure prediction tools, such as AlphaFold and Rosetta, have transformed structural biology by enabling accurate modeling of protein conformations and interactions, even in the absence of experimental data. Such tools can play a critical role in understanding the subtle structural interactions of ACE2 within the context of the RAS in the scope of DR.

### Using AlphaFold to resolve unknown structures

Much of the preceding work on the structural mechanics of ACE2 has been done in the context of its role as an infection vector for COVID-19. AlphaFold-predicted ACE2 conformations were used to validate structural models of ACE2 orthologous peptides before docking and molecular dynamics simulations, enabling the design of inhibitory peptides with enhanced binding affinity^90^. Similarly, AlphaFold has been used with ACE2 to gain a glimpse into the nature of its interaction with the SARS-CoV-2 spike protein, by generating protein structures of structurally unknown biological compounds to understand if the disruption of ACE2 binding could result in potentially harmful side effects in the host’s system^88^.

Beyond viral entry, these approaches can be extended to DR by modeling ACE2 variants, isoforms, or post-translational modifications that may shift enzymatic activity or binding affinity for Ang2/Ang-(1–7)^112–114^. AlphaFold can prioritize ACE2 complex partners and potential interface clashes, particularly when filtered by retina single-cell data^115^ or proteomics to focus on tissue-relevant interactors^116^. Coupling AlphaFold-informed docking with molecular dynamics then guides the rational design of peptides, small molecules, or even microbial metabolites that stabilize ACE2’s protective function while avoiding disruption of native interactions^117^.

### Validating predicted protein structures

Rosetta, a popular computational suite for macromolecular modeling, complements AlphaFold by refining structural predictions, exploring conformational landscapes, and performing energy-based docking and design^118^. On its own, Rosetta has been successfully used for ACE2 to create soluble receptor variants with improved binding affinity to the SARS-CoV-2 spike protein^119^ and ACE2-derived peptides that can act as competitive inhibitors^120^. More recently, hybrid pipelines that combine AlphaFold’s structural accuracy with Rosetta’s energetic refinement and flexible docking capabilities have enhanced the modeling of protein–protein interactions and ligand docking^121^.

### Integrating structural prediction with validation

Integrating Rosetta with AlphaFold in the context of DR can be used to improve AlphaFold-predicted models of ACE2 and its interactors, resample docking poses to account for conformational changes, and design peptides or small molecules that maintain ACE2’s protective Ang-(1–7) signaling cascade. This approach not only aids in identifying candidate therapeutics^118,121^, but also provides energy-based validation of ACE2’s structural stability under diabetic conditions, such as oxidative stress. By combining AlphaFold’s predictive power with Rosetta’s design tools, researchers can create a rational workflow for developing ACE2-targeted interventions that protect retinal tissues while minimizing disruption of ACE2’s natural biological interactions.

## Prospects

The multifaceted etiology of DR calls for innovative and comprehensive therapeutic strategies (see Box 7). Engineered probiotics designed to deliver therapeutic molecules offer a promising strategy to address systemic and retinal complications of diabetes. The gut microbiota’s pivotal role in modulating ACE2 expression highlights its potential as a therapeutic target for DR. Advances in probiotic engineering and metagenomics have made the precise manipulation of the microbiome increasingly feasible, paving the way for effective interventions.

Recent breakthroughs in computational biology have further boosted the potential of microbiome-based therapies. Within the next few years, it is realistic to expect these tools to yield significant clinical advancements. ML and DL have already revolutionized microbiome research by enabling sophisticated analyses of complex datasets. This will likely lead to validated predictive models that can identify microbial patterns to stratify patient risk for DR.

By analyzing baseline microbiome profiles alongside metagenomic and other omics data, AI systems can be refined to predict individual responses to probiotic treatments. This can guide the design of more targeted clinical trials (see Box 8). Moreover, the rise of explainable frameworks will be important in translating these complex computational predictions into clinical practice by making them interpretable to clinicians^122,123^.

Looking further ahead, the long-term vision for this field remains more speculative. Future directions emphasize hybrid computational models that incorporate microbiome, host genomic, and clinical data to provide a holistic understanding of host-microbiome interactions. The ultimate, though still distant, goal is to use these capabilities for truly personalized medicine and data-driven healthcare, where interventions are tailored to an individual’s unique biological landscape. However, several challenges must be addressed to fully realize this potential. Experimental validation of computational predictions remains a challenge, as does the gap between predictions and clinical reality. Many proposed interventions remain untested in animal models or early-phase trials, and discrepancies frequently emerge when computationally optimized strategies are applied in vivo. Bridging this divide will require standardized pipelines for validation, multi-omics datasets collected across diverse populations, and iterative feedback loops between computational modeling and experimental or clinical studies. Fostering interdisciplinary collaboration across microbiology, computational biology, and clinical sciences will be essential to overcoming these challenges. Also, ethical considerations related to data privacy must be prioritized to ensure responsible and equitable advancement of these transformative technologies.

The intersection of microbiome research, synthetic biology, and computational tools provides an exciting frontier for addressing the multifactorial challenges of DR. Through these innovations, we can move closer to holistic and personalized therapeutic solutions that benefit individuals with diabetes and its complications.

## Supplementary information

Supplementary Information

## Data Availability

No datasets were generated or analysed during the current study.
