# Supplementary material for: Systems biology and microbiome innovations for personalized diabetic retinopathy management
Source: NPJ Syst Biol Appl. 2025 Nov 21;11:133. doi: 10.1038/s41540-025-00607-w (PMC12638782; doi:10.1038/s41540-025-00607-w)
Supplement: Supplementary file 1 — Supplementary Information [file 41540_2025_607_MOESM1_ESM.pdf]

Systems biology and microbiome innovations for personalized diabetic retinopathy management

Supplementary Information

Javad Aminian-Dehkordi[1], Fateme Montazeri[2], Ali Tamadon[1], Mohammad R.K. Mofrad[1,3]*

1. Molecular Cell Biomechanics Laboratory, Departments of Bioengineering and Mechanical Engineering, University of California, Berkeley, California

2. Department of Ophthalmology & Vision Science, Tschannen Eye Institute, University of California, Davis, Sacramento, California

3. Molecular Biophysics and Integrative Bioimaging Division, Lawrence Berkeley National Lab, Berkeley, California

**Corresponding author: mofrad@berkeley.edu*

Table S1- Key factors associated with DR pathogenesis.

Key factor	Pathway name	Role in DR	Mechanism of action	Ref.
Renin-angiotensin system	Renin-angiotensin system	Promotes vascular dysfunction	Increases Angiotensin II levels, leading to endothelial dysfunction	[1]
Aldose reductase pathway	Polyol pathway	Osmotic stress and oxidative damage	Converts glucose to sorbitol, causing osmotic imbalance	[2]
Glucose transporter 1	Glucose transport pathway	Regulates glucose uptake in retinal cells	Facilitates cellular glucose transport under hyperglycemic conditions	[3]
Tumor necrosis factor-alpha	Inflammatory cytokine pathway	Induces inflammation and apoptosis	Activates inflammatory pathways *via* TNF-alpha receptors	[4]
Intercellular adhesion molecule-1	Cell adhesion pathway	Facilitates leukocyte adhesion and vascular damage	Binds leukocytes to endothelium, promoting vascular damage	[5]
Nitric oxide synthases	Nitric oxide signaling pathway	Regulates vascular tone and oxidative stress	Produces nitric oxide, regulating vascular function	[6]
Transforming growth factor-beta	TGF-beta signaling pathway	Mediates fibrosis and extracellular matrix changes	Stimulates fibroblast activation and collagen deposition	[7]
Hypoxia-inducible factor-1-alpha	Hypoxia response pathway	Enhances retinal hypoxia and neovascularization	Stabilizes HIF-1α in hypoxic conditions, driving VEGF expression	[8]
Receptor for advanced glycation end-products	AGE pathway	Leads to inflammation and oxidative stress	Forms AGEs that cross-link proteins and trigger receptor activation	[9]
Nuclear factor-kappa B	Inflammation and stress response pathway	Activates inflammatory cascades	Activates IKK complex, leading to pro-inflammatory gene transcription	[10]
Toll-like receptor 4	Innate immune response pathway	Initiates innate immune responses	Activates NF-κB and cytokine production	[11]
Interleukin-1	Pro-inflammatory cytokine pathway	Promotes inflammation and cell damage	Binds receptors, triggering downstream inflammatory pathways	[12]
C-reactive protein	Acute phase protein pathway	Increases systemic and local inflammation	Activates complement and inflammatory cascades	[13]
Monocyte chemoattractant protein-1	Monocyte chemoattractant signaling pathway	Attracts monocytes, promoting inflammation	Stimulates monocyte infiltration into inflamed tissue	[14]

Table S2- GEM integration tools for metabolic analysis

Method	Purpose	Algorithm	Core methodology	Unique features	Limitations	Ref.
GIMME	Integrates gene expression data to ensure model outputs are consistent with observed gene activity.	LP	Identifies the minimum set of reactions necessary to maintain consistency with gene expression data.	Focuses on excluding inconsistent reactions, making it efficient for small datasets.	Does not handle dynamic or proteomic data; less adaptable to noisy data.	[15]
TIGER	Uses gene expression and reaction relationships to refine metabolic flux predictions.	MILP	Encodes gene-protein-reaction (GPR) rules as constraints in GEMs, optimizing flux predictions through expression-level thresholds.	Flexibility in handling constraints derived from GPR rules; can model on/off gene activity.	Computationally intensive for large-scale problems; complex algorithms require expertise.	[16]
GEMsplice	Accounts for transcript isoform-level expression in metabolic pathway simulations.	trilevel LP	Matches isoform-level expression data to metabolic reactions, mapping spliced isoforms directly to pathway activity.	Highly granular analysis at the transcript level, ideal for precision medicine in transcript-related diseases.	Limited to transcriptomics data; not a general-purpose tool for metabolism-focused studies.	[17]
ICON-GEMs	Integrates gene co-expression networks into GEMs for pathway-level flux predictions.	QP	Uses co-expression matrices to infer regulatory constraints on fluxes, allowing for pathway-specific prioritization of gene activity.	Incorporates systemic, pathway-level regulatory effects into GEMs.	Assumes gene co-expression is directly tied to flux control, which may oversimplify dynamics.	[18]
PROSO Toolbox	Adds protein abundance constraints to GEMs, refining flux predictions.	bi-LP	Adds protein abundance as an additional layer of constraint, limiting flux predictions based on experimentally determined proteomics data.	Captures proteome-level impacts on fluxes, enabling protein-constrained GEM simulations.	Requires comprehensive proteomics data, which is not always available; high computational cost.	[19]

References

1. Lovshin JA, Lytvyn Y, Lovblom LE, Katz A, Boulet G, Bjornstad P, et al. Retinopathy and RAAS Activation: Results From the Canadian Study of Longevity in Type 1 Diabetes. Diabetes Care. 2019;42: 273–280.

2. Abhary S, Burdon KP, Laurie KJ, Thorpe S, Landers J, Goold L, et al. Aldose reductase gene polymorphisms and diabetic retinopathy susceptibility. Diabetes Care. 2010;33: 1834–1836.

3. Chen H, Zhang X, Liao N, Ji Y, Mi L, Gan Y, et al. Decreased expression of Glucagon-like peptide-1 receptor and Sodium-glucose co-transporter 2 in patients with proliferative diabetic retinopathy. Front Endocrinol (Lausanne). 2022;13: 1020252.

4. Morales-Lopez O, Rodríguez-Cortés O, López-Sánchez P, Pérez-Cano HJ, García-Liévanos O, Lima-Gómez V, et al. TNFα and IL-8 vitreous concentrations variations with two antidiabetic therapies in patients with proliferative diabetic retinopathy: an observational study. BMC Ophthalmol. 2024;24: 399.

5. Xie Z, Liang H. Association between diabetic retinopathy in type 2 diabetes and the ICAM-1 rs5498 polymorphism: a meta-analysis of case-control studies. BMC Ophthalmol. 2018;18: 297.

6. Khaloo P, Qahremani R, Rabizadeh S, Omidi M, Rajab A, Heidari F, et al. Nitric oxide and TNF-α are correlates of diabetic retinopathy independent of hs-CRP and HbA1c. Endocrine. 2020;69: 536–541.

7. Hirase K, Ikeda T, Sotozono C, Nishida K, Sawa H, Kinoshita S. Transforming growth factor beta2 in the vitreous in proliferative diabetic retinopathy. Arch Ophthalmol. 1998;116: 738–741.

8. Vezza T, Víctor VM. The HIF1α-PFKFB3 Pathway: A Key Player in Diabetic Retinopathy. J Clin Endocrinol Metab. 2021;106: e4778–e4780.

9. Ng ZX, Chua KH, Iqbal T, Kuppusamy UR. Soluble receptor for advanced glycation end-product (sRAGE)/pentosidine ratio: a potential risk factor determinant for type 2 diabetic retinopathy. Int J Mol Sci. 2013;14: 7480–7491.

10. Mitamura Y, Harada T, Harada C, Ohtsuka K, Kotake S, Ohno S, et al. NF-κB in epiretinal membranes after human diabetic retinopathy. Diabetologia. 2003;46: 699–703.

11. Wang L, Wang J, Fang J, Zhou H, Liu X, Su SB. High glucose induces and activates Toll-like receptor 4 in endothelial cells of diabetic retinopathy. Diabetol Metab Syndr. 2015;7: 89.

12. Coughlin BA, Christian B, Trombley B, Mohr S. Interleukin-1 receptor-dependent and -independent caspase-1 activity in retinal cells mediated by receptor interacting protein 2. Front Cell Dev Biol. 2024;12: 1467799.

13. Peng D, Wang J, Zhang R, Tang S, Jiang F, Chen M, et al. C-reactive protein genetic variant is associated with diabetic retinopathy in Chinese patients with type 2 diabetes. BMC Endocr Disord. 2015;15: 8.

14. Taghavi Y, Hassanshahi G, Kounis NG, Koniari I, Khorramdelazad H. Monocyte chemoattractant protein-1 (MCP-1/CCL2) in diabetic retinopathy: latest evidence and clinical considerations. J Cell Commun Signal. 2019;13: 451–462.

15. Blazier AS, Papin JA. Integration of expression data in genome-scale metabolic network reconstructions. Front Physiol. 2012;3: 299.

16. Jensen PA, Lutz KA, Papin JA. TIGER: Toolbox for integrating genome-scale metabolic models, expression data, and transcriptional regulatory networks. BMC Syst Biol. 2011;5: 147.

17. Angione C. Integrating splice-isoform expression into genome-scale models characterizes breast cancer metabolism. Bioinformatics. 2018;34: 494–501.

18. Paklao T, Suratanee A, Plaimas K. ICON-GEMs: integration of co-expression network in genome-scale metabolic models, shedding light through systems biology. BMC Bioinformatics. 2023;24: 492.

19. Yao H, Yang L. PROSO Toolbox: a unified protein-constrained genome-scale modelling framework for strain designing and optimization. arXiv [q-bio.MN]. 2023. Available: http://arxiv.org/abs/2308.14869
